# Yoga-Based Somatic Avatar Training for Alexithymia: Improving Distress Tolerance in College Students

**DOI:** 10.21203/rs.3.rs-11415292/v1

**Published:** 2026-10-20

**Authors:** Ellie Reznicek, Ashlin Ondrusek, Weyne Wang, Dennis McChargue

## Abstract

Introduction The difficulty of identifying and describing feelings (alexithymia) has been associated with problematic coping strategies (e.g. drinking) in college students. Expanding on prior work, the present study explored whether a novel two-week Somatic Avatar (SA) training would improve emotion regulation in college students and if the effect differed between students with varied levels of alexithymia. Method Sixteen college students ( *M*_age_ = 22.88; 81% non-Hispanic White) completed a two-week protocol that entailed four days of yoga training paired with three days of processing an individualized and animated avatar of their yoga practice. Participants completed demographic questions, the Multidimensional Assessment of Interoceptive Awareness scale (MAIA-2), and the Toronto Alexithymia Scale – 20 (TAS – 20) at baseline. They also completed baseline and exit assessments of the Distress Tolerance Scale (DTS). Median split groupings fell naturally in the moderate and high ranges of the TAS-20. Findings After controlling for interoception, a mixed-model analysis of covariance (ANCOVA) showed a significant effect of alexithymia on distress tolerance change scores from pre- to post- SA training, with participants endorsing moderate alexithymia ( *p* = .006) demonstrating greater improvement in distress tolerance than participants with high alexithymia ( *p* = .589), *F* (1, 13) = 6.346, *p* = .026, partial η2 = .328. Discussion Results suggested that the novel SA training may improve distress tolerance in those who struggle to identify their emotions. Future research should experimentally manipulate the length of training and increase the sample size to assess the effects of training among individuals with high alexithymia.

